# Impact of Sevoflurane and Thiopental Used Over the Course of Electroconvulsive Therapy: Propensity Score Matching Analysis

**DOI:** 10.3389/fnhum.2022.933622

**Published:** 2022-07-08

**Authors:** Taisuke Yatomi, Takahito Uchida, Akihiro Takamiya, Masataka Wada, Shun Kudo, Kazuki Nakajima, Hana Nishida, Bun Yamagata, Masaru Mimura, Jinichi Hirano

**Affiliations:** ^1^Department of Neuropsychiatry, Keio University School of Medicine, Tokyo, Japan; ^2^Department of Psychiatry, Melbourne Neuropsychiatry Centre, The University of Melbourne, Parkville, VIC, Australia

**Keywords:** anesthesia, electroconvulsive therapy, depression, sevoflurane, thiopental

## Abstract

**Objective:**

Although anesthetics play an important role in electroconvulsive therapy (ECT), the clinical efficacy and seizure adequacy of sevoflurane in the course of ECT remain unclear. The purpose of this study was to examine the clinical efficacy and seizure adequacy of sevoflurane, compared with those of thiopental, in the course of ECT in patients with mood disorders.

**Methods:**

We conducted a retrospective chart review. Patients who underwent a course of ECT and received sevoflurane (*n* = 26) or thiopental (*n* = 26) were included. Factors associated with ECT and treatment outcomes were compared between the two groups using propensity score (PS) matching. Between-group differences were examined using an independent *t*-test for continuous variables and a χ^2^-test for categorical variables.

**Results:**

Patients who received sevoflurane needed more stimulations (sevoflurane: 13.2 ± 4 times, thiopental: 10.0 ± 2.5 times, *df* = 51, *p* = 0.001) and sessions (sevoflurane: 10.0 ± 2.1 times, thiopental: 8.4 ± 2.1 times, *df* = 51, *p* = 0.01) and had more inadequate seizures (sevoflurane: 5 ± 3.9 times, thiopental: 2.7 ± 2.7 times, *df* = 51, *p* = 0.015). Remission and response rates were similar in both groups.

**Conclusion:**

The present findings indicate that sevoflurane should be used with caution in ECT and only when the clinical rationale is clear.

## Introduction

Major depressive disorder (MDD) is a common and debilitating psychiatric disorder and a leading cause of disability worldwide (Vigo et al., 2016). Despite a range of pharmacological treatments, 30% of patients show poor response (Rush et al., 2006) and are referred to as having treatment-resistant MDD. This group of patients may benefit from electroconvulsive therapy (ECT) (Weiss et al., 2019). ECT requires general anesthesia (Weiss et al., 2019), which affects seizure adequacy that may contribute to the clinical efficacy and tolerability of ECT (Kadiyala and Kadiyala, 2017). Intravenous anesthesia is commonly used in ECT. However, inhalational anesthesia would be preferable, specifically for patients with needle phobia, poor tolerance of intravenous induction agents, and agitation that affects their ability to cooperate (Rasmussen et al., 2005; Bryson et al., 2017; Erdil et al., 2017). Sevoflurane is the most common inhalational anesthetic used in ECT (Bryson et al., 2017; Aoki et al., 2021) because of its rapid and smooth induction (Loughnan et al., 2004). In addition, higher doses of sevoflurane may induce epileptiform electroencephalogram (EEG) (Gibert et al., 2012), which may improve seizure adequacy. Meanwhile, sevoflurane increases the expression level of gamma-aminobutyric acid-A receptors in the brain (Murao et al., 2002) and shortens seizure duration induced by ECT (Rasmussen et al., 2006; Aoki et al., 2021). These factors may negatively affect seizure adequacy. Previous studies have investigated the effect of sevoflurane on ECT using a crossover design (Calarge et al., 2003; Wajima et al., 2003; Hodgson et al., 2004; Loughnan et al., 2004; Rasmussen et al., 2005, 2006, 2007; Toprak et al., 2005; Matsubara et al., 2012; Begeç et al., 2013; Jadeja et al., 2014; Ulusoy et al., 2014; Erdil et al., 2015a,b, 2017; Pekel et al., 2016; Aoki et al., 2021). However, to the best of our knowledge, no previous study has investigated the effects of sevoflurane used throughout the course of ECT (i.e., when sevoflurane was used in all sessions of the course of ECT).

At our institution, intravenous anesthesia using thiopental (Nuzzi et al., 2018; Weiss et al., 2019) has been the standard protocol for ECT. However, sevoflurane is becoming increasingly popular as a standard because it facilitates rapid and smooth anesthesia induction. Therefore, we were able to compare the efficacy and outcomes associated with both anesthetics with minimal selection bias. This study aimed to examine the outcomes of sevoflurane used throughout the course of ECT, including seizure adequacy, and to compare them to those of thiopental use, using propensity score (PS) matching.

## Materials and Methods

The data used in this study included a retrospectively collected dataset from the Keio Neuropsychiatry ECT Database. Therefore, informed consent was not required from the patients. The database includes patients who received ECT for an MDD episode at the Keio University Hospital, Tokyo, Japan. This study included patients undergoing treatment between April 2012 and March 2019. This study was approved by the Ethics Committee of the Keio University School of Medicine and adhered to the tenets of the Declaration of Helsinki.

### Eligibility Criteria

Patients were included in this study if they received ECT for an MDD episode (including bipolar depression) at the Keio University Hospital, had a diagnosis of MDD or bipolar disorder based on the Diagnostic Statistical Manual of Mental Disorders (DSM) IV-TR or DSM-5, and received thiopental or sevoflurane for general anesthesia during ECT. To ensure the validity of the diagnosis, more than two psychiatrists (AT, TU, and HJ) evaluated the diagnosis of all the cases retrospectively. If the same patient underwent more than one course of ECT during the study period, the data from the first ECT course were used for the analysis.

Patients were excluded from this study if the anesthetic agent was changed over the course of ECT and if they underwent fewer than five ECT sessions.

### Participant Characteristics

Data on the participants’ demographic and clinical characteristics were extracted from our database, including age, sex, diagnosis, years of education, age at diagnosis, number of episodes, height, and weight.

### Clinical Assessments

The baseline severity of MDD was assessed using the Clinical Global Impression–Severity scale (CGI-S). The CGI-S is a seven-point scale, where a score of 1 point indicates a normal state and a score of 7 points indicates severe illness (Guy, 1976). In addition, psychotic, melancholic, and catatonic features, as defined by DSM IV-TR or DSM-5, were assessed at baseline.

Response to ECT was assessed using the clinical note Global Impression Improvement scale (c-CGI) (Kaster et al., 2017), which is a 4-point scale, where 1 point indicates excellent response and 4 points indicate poor response. This scale has been used in previous retrospective ECT chart reviews (Kristensen et al., 2013; Kaster et al., 2017). Three board-certified psychiatrists (AT, TU, and HJ) retrospectively assessed the CGI scores and other clinical features based on medical records; the assessors were blinded to the type of anesthetic used; any disagreements were resolved by discussion. Remission was defined as a c-CGI score of 1 point, and treatment response was defined as a c-CGI score of 1 or 2 points; therefore, patients who met the remission criteria also met the response criteria.

### Drug Dosage

The Anatomical Therapeutic Chemical (ATC) classification system and Defined Daily Dose (DDD) are recommended by the World Health Organization as measuring units for drug utilization studies.^1^ Based on the ATC classification, we identified hypnotics (ATC code: N05C), antidepressants (ATC code: N06A), anxiolytics (ATC code: N05B), antipsychotics (ATC code: N05A), anti-epileptics (ATC code: N03), and mood stabilizers (ATC code: N05AN). DDD is defined as an assumed average daily maintenance dose of a drug in a typical adult. When a patient receives two or more drugs, a summed DDD value is calculated per category.

### Electroconvulsive Therapy Protocol and Associated Factors

At our hospital, modified brief-pulse (0.5 ms) ECT (Thymatron system IV devices; Somatics, Inc., Lake Bluff, Ill) was administered twice or thrice weekly to inpatients. Bitemporal ECT using the half-age method was selected for all patients during the study period. When the initial stimulation did not cause adequate seizures based on the clinical judgment of the attending psychiatrist, the stimulation intensity was increased, and the patient received up to three more stimulations.

Treatment duration was determined by the clinical outcome and possible adverse events (Weiss et al., 2019). Specifically, ECT sessions were continued until symptomatic improvement reached a plateau, and when the last two sessions did not yield any further improvement according to the judgment of the psychiatrist-in-charge.

The following ECT-related factors were evaluated: number of ECT sessions per course (referred to as “number of ECT sessions”), number of stimulations per ECT course (referred to as “number of ECT stimulations”), charge used at the last ECT session, average charge used for ECT stimulations, average post-ictal suppression index, average seizure length, and number of inadequate seizures (defined as a seizure lasting < 20 s on EEG).

### Choice of Anesthetic

Sodium thiopental (3–5 mg/kg) or sevoflurane (8%) was used for general anesthesia, and succinylcholine (0.5–1.0 mg/kg) was used to induce muscle relaxation. The choice of anesthetic was determined by the attending anesthetists based on clinical standards (thiopental before 2016 and sevoflurane thereafter).

### Data Analysis

Participants’ characteristics were reported as descriptive statistics. Patients were divided into the sevoflurane and thiopental groups, and between-group differences were examined using an independent *t*-test for continuous variables and a χ^2^-test for categorical variables. All analyses were two-sided with an α-level set at 0.05. Data were analyzed using R Version 3.4.3.

### Propensity Score Matching

To control for confounding factors, we used PS matching analysis. PS matching reduces bias due to confounding factors by matching patients on baseline variables using a multivariable logistic regression model. PS matching was performed on the following factors: weight, height, and medications (DDD values of hypnotics, antidepressants, anxiolytics, antipsychotics, antiepileptics, and mood stabilizers), diagnosis of MDD or bipolar disorder (Bahji et al., 2019), CGI scores (van Diermen et al., 2018), presence of psychotic symptoms (van Diermen et al., 2018), and demographic characteristics such as age (van Diermen et al., 2018) and sex. PS-matched pairs were created at a ratio of 1:1 based on the nearest neighbor matching algorithm with a 0.20-caliper distance with no replacements. The goodness-of-fit of the logistic regression model was evaluated by the area under the curve. All PS matching procedures were conducted using the Matching package in R version 3.4.3.

## Results

**FIGURE 1:**
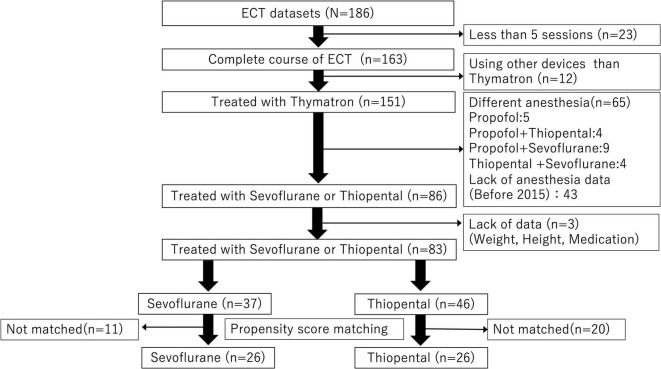
Data extraction flow. Among 186 patients with depression, 83 patients exposed to general anesthesia with sevoflurane or thiopental were included in the analysis. A total of 52 patients (26 patients per anesthetic group) were compared using propensity score matching.

Among 186 patients with MDD included in the database, 83 received sevoflurane or thiopental for all ECT sessions and were included in the present analysis. A total of 52 patients (26 patients per group) were compared using PS matching (Figure 1).

### Participant Demographics and Clinical Characteristics

Demographic and clinical characteristics of the overall sample are presented in Table 1. A total of 37 and 46 patients were included in the sevoflurane and thiopental groups, respectively. The demographic and clinical characteristics of both groups were similar. The sevoflurane group received more ECT stimulations (sevoflurane: 12.9 ± 4.0 times, thiopental: 10.8 ± 2.8 times, *df* = 82, *p* = 0.006), experienced more inadequate seizures (sevoflurane: 5.2 ± 4.3 times, thiopental: 2.9 ± 2.9 times, *df* = 82, *p* = 0.006), and received a higher average charge (sevoflurane: 296.0 ± 110.0 mc, thiopental: 247.9 ± 84.6 mc, *df* = 82, *p* = 0.032) than the thiopental group.

**TABLE 1:** Participant characteristics before propensity score matching.

	Sevoflurane			Thiopental			SMD	*p*
		
	(*n* = 37)			(*n* = 46)				
Age (years)	63.32	±	14.46	61.65	±	14.14	0.117	0.597
Sex, males n (%)	16		(43.2)	18		(39.1)	0.084	0.877
Diagnosis, MDD n (%)	32		(86.5)	37		(80.4)	0.163	0.662
Education years (years)	14.51	±	2.1	13.83	±	2.51		0.187
Onset age (years)	55.89	±	17.64	52.39	±	17.98	0.197	0.377
Duration of disease (months)	80.49	±	98	105.72	±	123.88		0.315
Number of episodes (times)	2.41	±	1.82	2.59	±	2.77		0.732
Duration of the current episode (months)	16.12	±	44.67	20.83	±	36.93	0.115	0.601
Psychotic symptoms, n (%)	13		(35.1)	20		(43.5)	0.171	0.585
Height (cm)	160.75	±	9.6	160.1	±	8.15	0.073	0.738
Weight (kg)	52.9	±	12.81	52.69	±	12.42	0.016	0.941
Number of ECT sessions (times)	9.59	±	2.35	8.96	±	2.17		0.203
Number of total stimulation (times)	12.89	±	3.97	10.78	±	2.82		0.006**
Inadequate seizures (times)	5.16	±	4.26	2.93	±	2.9		0.006**
Last ECT charge (mc)	361.67	±	134.42	337.13	±	136.08		0.416
Average ECT charge (mc)	295.95	±	109.97	247.92	±	84.57		0.032*
Average ECT GTC seizures (seconds)	43.33	±	10.16	44.31	±	8.69		0.636
Average ECT PSI (%)	74.72	±	11.72	78.75	±	11.9		0.132
Antidepressants (DDD)	1.11	±	0.83	0.9	±	0.94	0.237	0.29
Antipsychotics (DDD)	0.2	±	0.27	0.34	±	0.43	0.39	0.088
Anxiolytics (DDD)	0.06	±	0.23	0.07	±	0.26	0.044	0.845
Hypnotics (DDD)	0.78	±	0.73	0.61	±	0.83	0.219	0.328
Anti-epileptic drug (DDD)	0	±	0.01	0.01	±	0.06	0.305	0.192
Mood stabilizer (DDD)	0	±	0	0.1	±	0.71	0.209	0.373
Pre CGI-7	4.84	±	1.21	4.63	±	1.36	0.161	0.47
Post CGI	1.56	±	0.89	1.78	±	1		0.316
CGI remission, n (%)	22		(64.7)	24		(53.3)		0.433
CGI response, n (%)	29		(85.3)	35		(77.8)		0.58

*Values are expressed as mean ± SD unless otherwise indicated. The between-group differences were examined using the independent t-test for continuous variables and the χ^2^-test for categorical variables.*

SMD, Standardized Mean Difference; MDD, major depressive disorder; ECT, electroconvulsive therapy; GTC, generalized tonic-clonic seizures; PSI, Post-Ictal Suppression index; DDD, defined daily dose; CGI, Clinical Global Impressions. *p < 0.05, **p < 0.01.

### Results of Propensity Score-Matched Analysis

**FIGURE 2:**
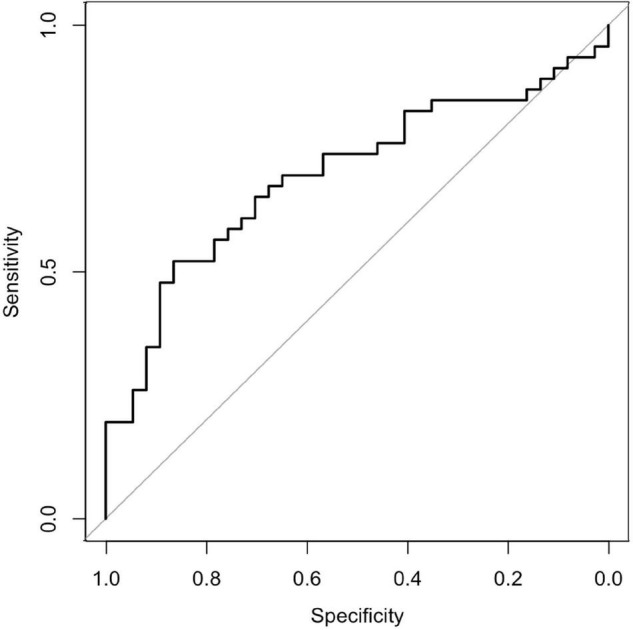
Receiver operating characteristic curve. The area under the receiver operating characteristic curve was 0.696 (95% confidence interval: 0.582–0.81).

**FIGURE 3:**
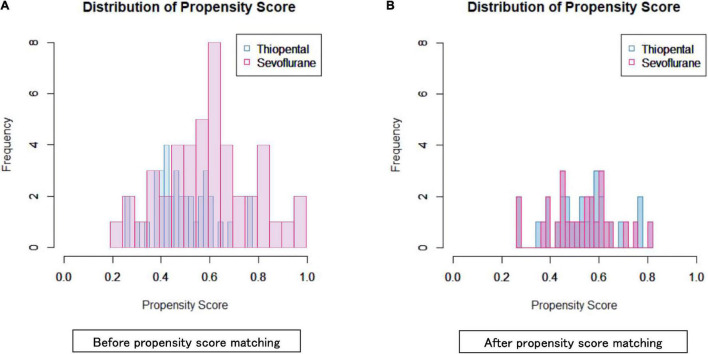
Propensity score distribution in the sevoflurane and thiopental groups before **(A)** and after **(B)** propensity score matching.

Although the area under the receiver operating characteristic curve was 0.696 (95% confidence interval: 0.582–0.811) (Figures 2, 3), PS matching showed that the distribution of PS was well matched in the study. Demographic and clinical characteristics were similar between the PS-matched sevoflurane and thiopental groups. The sevoflurane group required more ECT stimulations (sevoflurane: 13.2 ± 4.0 times, thiopental: 10.0 ± 2.5 times, *df* = 51, *p* = 0.001), more ECT sessions (sevoflurane: 10.0 ± 2.1 times, thiopental: 8.4 ± 2.1 times, *df* = 51, *p* = 0.01), and experienced more inadequate seizures (sevoflurane: 5.0 ± 3.9 times, thiopental: 2.7 ± 2.7 times, *df* = 51, *p* = 0.015) than the thiopental group. The characteristics of the PS-matched participants are presented in Table 2.

**TABLE 2:** Participant characteristics after propensity score matching.

	Sevoflurane			Thiopental			SMD	*p*
		
	(*n* = 26)			(*n* = 26)				
Age (years)	63.31	±	13.64	63.46	±	13.78	0.011	0.968
Sex, males n (%)	11		(42.3)	10		(38.5)	0.078	1
Diagnosis, MDD n (%)	21		(80.8)	23		(88.5)	0.214	0.701
Education years (years)	14.42	±	2.16	14.04	±	2.57		0.561
Onset age (years)	55	±	17.98	52.77	±	19.57	0.119	0.67
Duration of disease (months)	87.62	±	102.19	115.38	±	144.14		0.427
Number of episodes (times)	2.62	±	1.96	2.31	±	1.81		0.559
Duration of current episode (months)	9.04	±	8.27	25.85	±	42.59	0.548	0.054
Psychotic symptoms, n (%)	9		(34.6)	10		(38.5)	0.08	1
Height (cm)	160.47	±	9.79	160.72	±	9.31	0.026	0.925
Weight (kg)	51.62	±	12.84	53.07	±	13.5	0.11	0.694
Number of ECT sessions (times)	9.96	±	2.09	8.42	±	2.06		0.01**
Number of total stimulation (times)	13.19	±	4	10.04	±	2.54		0.001**
Inadequate seizures (times)	5	±	3.93	2.65	±	2.67		0.015*
Last ECT charge (mc)	360.56	±	140.92	325.68	±	120.96		0.343
Average ECT charge (mc)	296.7	±	45.12	252.7	±	79.18		0.112
Average ECT GTC seizures (seconds)	42.43	±	8.24	43.64	±	7.38		0.581
Average ECT PSI (%)	75.39	±	12.23	80.08	±	10.2		0.15
Antidepressants (DDD)	1.1	±	0.9	0.9	±	0.96	0.22	0.431
Antipsychotics (DDD)	0.23	±	0.3	0.16	±	0.25	0.251	0.369
Anxiolytics (DDD)	0.05	±	0.24	0.11	±	0.33	0.219	0.433
Hypnotics (DDD)	0.66	±	0.73	0.65	±	0.87	0.016	0.955
Anti-epileptic drug (DDD)	0	±	0.01	0	±	0	0.277	0.322
Mood stabilizer (DDD)	0	±	0	0	±	0	< 0.001	NaN
Pre CGI-7	4.81	±	1.2	4.5	±	1.48	0.229	0.414
Post CGI	1.6	±	0.96	1.65	±	0.89		0.836
CGI remission, n (%)	16		(64.0)	15		(57.7)		0.862
CGI response, n (%)	21		(84.0)	21		(80.8)		1

*Values are expressed as mean ± SD unless otherwise indicated. The between-group differences were examined using an independent t-test for continuous variables and a χ^2^-test for categorical variables.*

*SMD, Standardized Mean Difference; MDD, major depressive disorder; ECT, electroconvulsive therapy; GTC, generalized tonic-clonic seizures; PSI, Post-ictal Suppression Index; DDD, defined daily dose; CGI, Clinical Global Impressions; NaN.*

*p < 0.05, **p < 0.01, ***p < 0.005.

## Discussion

This study examined the clinical efficacy and seizure adequacy associated with ECT in patients with MDD who received sevoflurane or thiopental throughout the ECT course. In the present study, the number of stimulations, ECT sessions, and inadequate seizures were significantly higher in the sevoflurane group than in the thiopental group. These findings suggest some disadvantages of the routine use of sevoflurane as a general anesthetic for ECT.

ECT is a treatment option for the most severe psychiatric disorders, making it difficult to conduct a randomized controlled trial on the impact of the anesthetic type used during treatment administration (Fond et al., 2016); consequently, we used PS matching in this study. Previous studies on the use of sevoflurane in ECT involved crossover or parallel randomized designs (Calarge et al., 2003; Wajima et al., 2003; Hodgson et al., 2004; Loughnan et al., 2004; Toprak et al., 2005; Rasmussen et al., 2006, 2007; Matsubara et al., 2012; Begeç et al., 2013; Jadeja et al., 2014; Ulusoy et al., 2014; Erdil et al., 2015a,b, 2017; Pekel et al., 2016; Aoki et al., 2021). To the best of our knowledge, this is the first study to investigate the impact of sevoflurane used during the entire treatment course on the clinical efficacy and seizure adequacy of ECT. This study involved a proper evaluation of matching (Ali et al., 2015), and the distribution of PS overlapped between the groups and covariates was balanced (Bergstra et al., 2019).

Aoki et al. (2021) performed a meta-analysis showing that sevoflurane shortened seizure duration and increased heart rate compared with other intravenous anesthetics. In addition, previous cross-over studies on the clinical effects of sevoflurane and thiopental (Rasmussen et al., 2006; Jadeja et al., 2014) showed that sevoflurane shortened seizure duration compared with thiopental. Although the duration of seizures was similar in the sevoflurane and thiopental groups in the present study, the frequency of inadequate seizures was higher in the sevoflurane group than in the thiopental group. The increase in the number of inadequate seizures associated with sevoflurane may have clinical implications. In fact, in our dataset, the sevoflurane group received more ECT sessions and ECT stimulations than the thiopental group. These increases may result in increased hospitalization duration and financial burden to both patients and healthcare systems. Furthermore, the number of stimulations has been associated with the risk of delirium (Tsujii et al., 2019), which is an adverse neuropsychological event. A previous study showed that the number of treatments was relevant to cognitive decline in specific cognitive domains such as autobiographical memory (Sackeim et al., 2007; Porter et al., 2008). Relevant to this evidence, increasing the number of treatments in the sevoflurane group may exacerbate cognitive decline caused by ECT. Moreover, ECT causes generalized autonomic nervous system response, with initial parasympathetic-induced bradycardia followed immediately by a sympathetic response that results in transient tachycardia and hypertension (Ding and White, 2002; Uppal et al., 2010) and myocardial oxygen consumption increases (Ding and White, 2002; Uppal et al., 2010). An increased number of sessions may increase the occurrence of arrhythmias and cardiac overloads, which may be problematic, particularly in older adults.

In the present study, the sevoflurane and thiopental groups achieved similar treatment outcomes; this finding is consistent with that of a previous study (Fond et al., 2016), which concluded that the evidence to recommend a specific anesthetic for ECT is insufficient, and the choice should be at the discretion of the attending physician, given the adverse event profile and seizure duration. This approach makes room for the use of sevoflurane in this context (Andrade, 2021). Sevoflurane may be useful for patients who are agitated or afraid of injections (Rasmussen et al., 2005), experience problematic prolonged seizures (Andrade, 2021), and are pregnant, as this agent helps reduce uterine contractions after ECT (Ishikawa et al., 2001; Andrade, 2021).

This study had some limitations. First, data were collected retrospectively, including information on treatment outcomes. Specifically, the retrospective use of CGI-S and c-CGI may attenuate the reliability of treatment outcomes. Future research may need a structured assessment of symptoms. Second, the time interval between anesthetic and ECT administrations and depth of anesthesia were not evaluated; all of these factors may affect seizure quality (Kranaster et al., 2013; Gálvez et al., 2016; Gálvez and Loo, 2017; Taylor et al., 2019). Consequently, we could not exclude the possibility that our results reflect differences in the depth of anesthesia rather than differences in the action of anesthetic agents. Third, we might not completely eliminate the other confounding factor that potentially affects the convulsion’s quality.

The present findings suggest potential disadvantages of sevoflurane for ECT, as it may increase the number of ECT stimulations and sessions required and that of inadequate seizures experienced by patients. The present findings suggest that sevoflurane for ECT should be used cautiously and only when the clinical rationale is clear.

## Data Availability Statement

The datasets presented in this article are not readily available because participants of this study did not consent to their data being shared publicly. Requests to access the datasets should be directed to the corresponding author/s.

## Ethics Statement

The studies involving human participants were reviewed and approved by the Ethics Committee of the Keio University School of Medicine. Written informed consent for participation was not required for this study in accordance with the national legislation and the institutional requirements.

## Author Contributions

TY, TU, and JH were involved in the analysis, design, and interpretation of the data, wrote the first draft of the manuscript, and supervised the entire project. TY, TU, AT, MW, SK, KN, HN, BY, MM, and JH contributed to the interpretation of the data. All authors contributed to the article and approved the final manuscript.

## Conflict of Interest

The authors declare that the research was conducted in the absence of any commercial or financial relationships that could be construed as a potential conflict of interest.

## Publisher’s Note

All claims expressed in this article are solely those of the authors and do not necessarily represent those of their affiliated organizations, or those of the publisher, the editors and the reviewers. Any product that may be evaluated in this article, or claim that may be made by its manufacturer, is not guaranteed or endorsed by the publisher.

## Footnotes

1
http://www.whocc.no/atc_ddd_index/

## Abbreviations

ATC: Anatomical Therapeutic Chemical
c-CGI: clinical note Clinical Global Impression scale
CGI-S: Clinical Global Impression–Severity scale
DDD: Defined Daily Dose
DSM: Diagnostic Statistical Manual of Mental Disorders
ECT: electroconvulsive therapy
EEG: electroencephalogram
MDD: major depressive disorder
PS: propensity score.
